# Intercellular transfer of HLA‐G: its potential in cancer immunology

**DOI:** 10.1002/cti2.1077

**Published:** 2019-08-30

**Authors:** Aifen Lin, Wei‐Hua Yan

**Affiliations:** ^1^ Biological Resource Center Taizhou Hospital of Zhejiang Province Wenzhou Medical University Linhai Zhejiang China; ^2^ Medical Research Center Taizhou Hospital of Zhejiang Province Wenzhou Medical University Linhai Zhejiang China

**Keywords:** cancer, exosome, HLA‐G, trogocytosis, tunnelling nanotubes

## Abstract

Intercellular protein transfer between cancer cells and immune cells is a very common phenomenon that can affect different stages of host antitumor immune responses. HLA‐G, a non‐classical HLA class I antigen, has been observed to be widely expressed in various malignancies, and its immune‐suppressive functions have been well recognised. HLA‐G expression in cancer cells can directly mediate immune tolerance by interacting with inhibitory receptors such as ILT2 and ILT4 expressed on immune cells. Moreover, a network of multiple directional intercellular transfers of HLA‐G among cancer cells and immune cells through trogocytosis, exosomes and tunnelling nanotubes provides malignant cells with an alternative ploy for antigen sharing and induces more complex heterogeneity, to modulate immune responses, ultimately leading to immune evasion, therapy resistance, disease progression and poor clinical outcome. Herein, we discuss the relative aspects of the intercellular transfer of HLA‐G between tumor cells and immune cells and its potential use in tumor immunology research and translational cancer therapy.

## Introduction

The host innate and adaptive immune system is one of the most obvious hallmarks of cancers.^1^ To this end, tumor cells have developed various direct and indirect mechanisms to impair the functions of immune cells in tumor microenvironments for their persistence and survival. In this scenario, aberrant induction of HLA‐G expression in malignant cells represents one of the key factors that contributes to tumor immune escape and progression.^2^

HLA‐G is a member of the non‐classical HLA antigens that was first discovered on extravillous cytotrophoblasts in 1990.^3^ Unlike other HLA counterparts, HLA‐G has distinctive features with limited nucleotide variations, peptide repertoire and tissue distribution. However, the molecular structure of HLA‐G is diverse. To date, at least seven HLA‐G isoforms can be generated by the alternative splicing of the primary HLA‐G mRNA. Among them, four membrane‐bound (HLA‐G1‐HLA‐G4) and three soluble (HLA‐G5‐HLA‐G7) isoforms have been well studied.^4^ However, more novel HLA‐G isoforms and their biological functions and clinical significance remain to be uncovered as addressed in recent studies.^5^, ^6^

Since it was first observed on the foetal–maternal interface of extravillous cytotrophoblasts, a broader aberrant induction of HLA‐G expression has been found in tissues and cells such as cancer cells, virus‐infected cells and transplant grafts.^7^ In addition, the functional properties of HLA‐G have been extensively explored both *in vitro* and *in vivo*.^8^ It has been widely acknowledged that HLA‐G is a potent immune inhibitory molecule that can interact with immune inhibitory receptors (particularly ILT2 and ILT4) expressed on various immune effectors.^9^ As a consequence, HLA‐G expression is favorable when it maintains the foetal–maternal immune tolerance and transplanted graft acceptance; however, it is harmful when it promotes immune evasion by cancer cells or virus‐infected cells.^7^

In the context of tumor immunology, HLA‐G can comprehensively impair host antitumor immune responses in multiple direct or indirect pathways.^10^ With the engagement of inhibitory receptors, HLA‐G expressed by malignant cells can directly impair the functions of different immune effectors such as NK cells, T cells, B cells, neutrophils, macrophages and dendritic cells (DCs). Moreover, HLA‐G–receptor interactions can also induce and promote the expansion of immune regulatory cells such as regulatory T cells (Tregs), tolerogenic DCs and myeloid‐derived suppressive cells (MDSCs).^2^ Consequently, HLA‐G expression in cancers has been frequently observed to be associated with disease progression and poor clinical outcome.^9^ However, intercellular transfer of tumor cell‐derived HLA‐G molecules through trogocytosis, exosomes and tunnelling nanotubes (TnTs) represents another important complementary mechanism for cancer cell escape from destruction by the host immune system.^11^, ^12^

Intercellular transfer of cell‐surface proteins, such as trogocytosis, exosomes and TnTs, is a very common phenomenon that can affect many different stages of immune responses in both physiological and pathological conditions.^13^ Intercellular transfer of tumor proteins has been observed to be associated with the various aspects of malignant behaviour of cancer cells and their surrounding microenvironment to promote cancer progression.^14^ By intercellular transfer of surface proteins, donor cancer cells can modulate the activity of recipient cells and play important roles in tumorigenesis, growth, metastasis and even drug resistance. Moreover, cancer cells using intercellular transfer of cell‐surface proteins also play critical roles in re‐shaping the tumor microenvironment to support their expansion and metastatic activity, as well as dampen antitumor immune responses.^15^, ^16^ In this context, a recent study by Hamieh *et al*.^17^ revealed that both CD19 and CD22 expressed on NALM6 acute lymphoblastic leukaemia cells could be transferred to chimeric antigen receptor (CAR)‐T cells through the process of trogocytosis. Consequently, the density of the target molecules CD19 and CD22 was decreased on the tumor cells, and the T‐cell killing activity was abated by the fratricide and exhaustion of the T cells with molecules acquired target by trogocytosis by CAR‐T cells. In a study of another tumor antigen, carcinoembryonic antigen (CEA), the authors presented that the killing ability of NK cells was inhibited when they rapidly and specifically acquired CEA molecules via trogocytosis from the CEA‐expressing 721.221 cells.^18^ In the context of tumor‐derived exosomes, the levels of exosomal programmed death‐ligand 1 (PD‐L1) have been intensively investigated. Studies have shown that levels of exosomal PD‐L1 are associated with tumor progression in different types of malignancies such as metastatic melanoma,^19^ glioblastoma,^20^ and head and neck and breast cancers.^21^, ^22^ Among various potential mechanisms, Poggio *et al*.^23^ recently noted that exosomal PD‐L1 could suppress draining lymph node T‐cell activity and enable cancer cells to escape antitumor immunity. However, the significance of intercellular communication by TnTs, first reported in 2004, remains less known.^24^ Fortunately, a rapidly increasing body of literature has revealed that TnTs could be formed among different cell types, such as various immune cells, epithelial cells and cancer cells, and further confirmed that TnT is an important pathway for intercellular exchanges of organelles, proteins, signals and pathogens.^25^ Depending on the TnTs formed among different cell types, TnTs can play critical roles in viral entry and dissemination, immune response regulation, tumor metastasis and drug resistance.^26^ Regarding cancer, Osswald *et al*.^27^ provided the first *in vivo* evidence of the functions of TnTs in a nude murine tumor‐bearing model with patient‐derived astrocytoma cells and showed that TnT‐connected astrocytoma cells could promote tumor progression and resistance to antitumor therapy.

**Figure 1:**
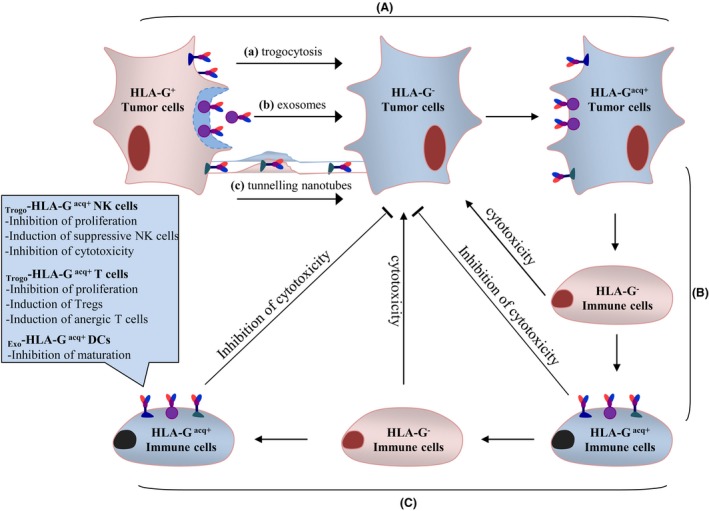
Immune suppression induced by intercellular transfer of tumor HLA‐G. (**A**) HLA‐G^−^ cancer cells can acquire HLA‐G molecules from HLA‐G^+^ cancer cells and become HLA‐G^acq+^ cancer cells via trogocytosis (**a**), exosomes (**b**) and tunnelling nanotubes (**c**). (**B**) Immune cells can acquire HLA‐G molecules from HLA‐G^+^ cancer cells and become HLA‐G^acq+^ immune cells, such as HLA‐G^acq+^ CD4^+^ T cells, HLA‐G^acq+^ CD8^+^ T cells, HLA‐G^acq+^ NK cells and HLA‐G^acq+^ CD14^+^ monocytes. (**C**) Immune cells can acquire HLA‐G molecules from HLA‐G^+^ immune cells and become HLA‐G^acq+^ immune cells such as HLA‐G^acq+^ CD4^+^ T cells, HLA‐G^acq+^ CD8^+^ T cells and HLA‐G^acq+^ monocytes. Immune cell functions are suppressed after acquiring HLA‐G via different intercellular transfer processes, such as the inhibition of proliferation and cytotoxicity, and induction of suppressive NK cells in HLA‐G^acq+^ NK cells; the inhibition of proliferation and induction of Treg and anergic T cells in HLA‐G^acq+^ T cells, and the impairment of the maturation of HLA‐G^acq+^ dendritic cells.

Indeed, a large body of research has reported that intercellular exchange of membrane proteins such as HLA‐G molecules occurs not only between tumor cells and immune cells but also between tumor cells and tumor cells.^28^, ^29^, ^30^ Consequently, the multidirectional exchange of HLA‐G can lead to functional interference of both immune cells and tumor cells.^2^, ^11^ Herein, we focus on the very aspects of the intercellular transfer of tumor cell‐derived HLA‐G by mechanisms such as trogocytosis, exosomes and nanotubes in tumor immune regulation (Figure 1).

## Intercellular transfer of HLA‐G by trogocytosis

Trogocytosis is a rapid process that takes place within an hour or even within minutes, and actively transfers cell‐surface membrane fragments containing various membrane‐anchored proteins from one cell to an acquirer cell in a strictly physical cell‐to‐cell contact‐dependent manner.^31^ The detailed initiation of intercellular protein transfer between donor and acceptor cells through trogocytosis remains to be explored, but likely triggered by antigen receptor signalling and formation of an immunologic synapse.^32^ Consequently, phenotype and biological function of the acquirer cell (e.g. immune cell) can be modified with the proteins (e.g. MHC antigens) taken up from the donor cells by trogocytosis.^33^ Intercellular transfer of MHC antigens between immune cells was first reported as early as 1972 showing that MHC II proteins expressed on B cells had been transferred to T cells.^34^ However, trogocytosis can be multidirectional depending on the contacted cells in the vicinity.^35^ Trogocytosis of HLA‐G from tumor cells to immune cells, from immune cells to immune cells and from tomor cells to tumor cells has been reported in previous studies, which have demonstrated comprehensive immune inhibitory effects favoring malignancy development, disease relapse and even a poor disease outcome.^28^, ^29^, ^30^

As early as 2002, Wiendl *et al*.^36^ reported that having <10% HLA‐G‐positive glioma cell lines is sufficient to suppress alloreactive CD4^+^ T cells and CD8^+^ T‐cell lysis of HLA‐G‐negative glioma cells. Using the NK‐92 cell line as effectors, we found that HLA‐G expression in more than 20% of K562 cells can significantly inhibit NK cell lysis.^37^ In this scenario, it seems possible that, on the one hand, few HLA‐G‐positive tumor cells may confer enough protective effects to HLA‐G‐negative tumor cells through the rapid transfer of HLA‐G by trogocytosis among the tumor cells. On the other hand, effector cells such as lymphocytes and NK cells that have acquired HLA‐G from targets by trogocytosis can temporarily turn into regulatory cells, further empowering the immune evasion of tumor cells.^38^, ^39^ Thus, the multidirectional trogocytosis of HLA‐G among effectors or among tumor cells constitutes an alternative tolerogenic function of HLA‐G in tumor immunology.

## Trogocytosis of HLA‐G from tumor cells to immune cells

Immune cells acquiring HLA‐G from tumor cells by trogocytosis have been demonstrated by LeMaoult *et al*.^38^ In that study, the authors reported that both peripheral resting and activated CD4^+^ and CD8^+^ T cells from healthy donors can uptake HLA‐G from HLA‐G‐transfected LCL‐721.221 cells (LCL‐HLA‐G1) or HLA‐G expressed in IFN‐γ‐treated peripheral monocytes by trogocytosis. Although the degree of HLA‐G expression between the donor cells (LCL‐HLA‐G‐ and HLA‐G‐expressing monocytes) was dramatically different, the levels of HLA‐G acquired by the T cells (HLA‐G^acq+^ T cells) were very comparable. Moreover, acquired HLA‐G can directly inhibit CD4^+^ T‐cell proliferation and response to IL‐2 stimulation, and CD4^+^HLA‐G^acq+^ T cells behaved as regulatory T cells that could impair the reactivity of autologous T cells. In clinical settings, Brown *et al*.^29^ demonstrated that T cells acquired HLA‐G *in vivo* from autologous multiple myeloma plasma cells and turned into acquired CD25^−^FoxP3^−^ regulatory cells (Treg^acq^). The authors indicated that the novel Treg^acq^ can render more complexity to the immune response defects in multiple myeloma or other malignancies.

For NK cells, the IL‐2‐activated NK cell line NKL can acquire HLA‐G1 from an HLA‐G‐transfected melanoma cell line (M8‐HLA‐G1).^39^ Consequently, the proliferation and cytolytic capability of the NKL‐HLA‐G1^acq+^ cells was dramatically impaired, and the NKL‐HLA‐G1^acq+^ cells became suppressive NK cells. In addition to the NKL‐HLA‐G1^acq+^ cells, the immune‐suppressive function can extend to other NK cell cytolytic functions through the receptor ILT2. However, the immune‐suppressive properties of NKL‐HLA‐G1^acq+^ do not last long. When the HLA‐G acquired by the NK cell was internalised and degraded, the cytotoxic capability of the NK cells could be recovered. Thus, the acquisition of HLA‐G molecules through trogocytosis was transient and reversible. In line with this, a cycle of HLA‐G acquisition–degradation–re‐acquisition via trogocytosis from foetal HLA‐G^+^ extravillous trophoblasts (EVT) by decidual NK (dNK) cells has been demonstrated.^40^ In a co‐culture system, one‐fourth of dNK cells can uptake HLA‐G from EVT (dNK^acq+^), and then, HLA‐G can be internalised by endocytosis with the receptor KIR2DL4. When dNK^acq+^ cells were activated with either IL‐15 or IL‐12, both the cell surface and the internalised intracellular HLA‐G could be lost paralleled by the dramatic increase in cytotoxicity of dNK cells (dNK^acq−^). Interestingly, the lost HLA‐G can be re‐acquired through trogocytosis when the activated dNK cells are co‐cultured with EVT again. Given that many similar features are shared between the EVT and cancer cells, such as the expression of HLA‐G and the potential for cell proliferation, migration and vascular formation,^41^ whether a similar trogocytic recycling of HLA‐G can be acquired by immune cells infiltrating the tumor microenvironmental from HLA‐G‐positive tumor cells remains to be explored.

Acquisition of HLA‐G from tumor cells seems to be more proficient than from NK cells and T cells; however, trogocytosis of HLA‐G to monocytes does not have an impact on their functions because of the transient nature of HLA‐G on the cell surface as compared to that on NK cells and T cells.^42^ This might be explained by the fact that the acquired HLA‐G was internalised much faster in monocytes than in NK cells and T cells.

## Trogocytosis of HLA‐G from tumor cells to tumor cells

Tumor cells are populations with high heterogeneity. Trogocytic exchanges of membrane patches containing cell‐surface proteins between surrounding tumor cells *in vivo* are relevant to the phenotypic and functional properties of the host cells.^43^ A study by LeMaoult *et al*.^30^ demonstrated that haematological tumor cell lines can uptake HLA‐G molecules from either allogeneic or autologous donors. In that study, the trogocytic capability of tumor cells from haematological malignancy patients *ex vivo* and in ten haematological tumor cell lines *in vitro* was investigated. Among liquid tumor cell lines, the data revealed that all cell lines such as histiocytic lymphoma (monocyte) U937 cells could acquire HLA‐G protein from autologous HLA‐G‐transfected U937 (U937‐HLA‐G) and allogeneic HLA‐G‐transfected LCL‐721.221 cells (LCL‐HLA‐G1). However, the trogocytic capabilities varied among cell lines, with U937 and Ramos cells ranking at the top among the cell lines investigated in the study. Furthermore, similar trogocytosis features were observed in freshly isolated cells from haematological malignancy patients. These data indicated that trogocytosis of HLA‐G can occur between haematopoietic malignant tumor cells or other malignant cells in the development of the disease.

## Trogocytosis of HLA‐G from immune cells to immune cells

Beyond the transfer of HLA‐G from tumor cells to immune cells, and from tumor cells to tumor cells, a multi‐step and serial trogocytosis has also been observed.^28^ In this scenario, monocytes that had acquired HLA‐G molecules from multiple tumor cell lines such as LCL‐HLA‐G1 and M8‐HLA‐G1 could further redistribute the acquired membranes to other recipients such as CD4^+^ T cells, CD8^+^ T cells and CD14^+^ monocytes. While studying other immune checkpoint molecules such as PD‐L1, Gary *et al*.^44^ noted that CD8^+^ T cells could take up the functionally active PD‐L1 molecules from Melan‐A‐pulsed mature DCs and result in apoptosis and fratricide of PD‐1‐expressing neighbouring T cells, which might have a negative impact on the immune responses in malignant diseases. Although evidence for membrane‐bound HLA‐G‐induced apoptosis of immune cells remains to be explored, soluble HLA‐G (sHLA‐G) has been observed to induce apoptosis of CD8^+^ T lymphocytes and CD8^+^ NK cells. The underlying mechanism for the involvement of sHLA‐G in the apoptosis of CD8^+^ immune cells depends on the Fas/sFasL interaction, which is activated by the α3 domain of sHLA‐G molecules binding to the CD8 expressed on the abovementioned immune cells.^45^ Similar effects were observed in NK cells isolated from peripheral blood lymphocytes, in which sHLA‐G could effectively inhibit NK lysis and induce fratricide killing of NK cells by apoptosis independent of the receptor ILTs. However, the interaction between sHLA‐G and CD8 could not be excluded as the author noted that one‐fourth of the NK cells were expressing CD8.^46^ Because the extracellular α3 domain in the HLA‐G heavy chain exists in isoforms, such as HLA‐G1, HLA‐G2, HLA‐G5, HLA‐G6 and novel isoforms expected by Tronik‐Le Roux and co‐workers,^5^ making it possible that the α3 domain‐containing HLA‐G isoforms have the potential to trigger apoptosis of CD8^+^ immune cells.

By the process of trogocytosis, on the one hand, very few HLA‐G‐positive tumor cells may rapidly generate a large number of HLA‐G‐positive cells, which can directly inhibit antitumor immune responses through the interaction with the immune inhibitory receptor ILTs. On the other hand, immune cells acquiring HLA‐G from tumor cells or other immune cells by direct, multiple and/or serial patterns can turn into regulatory cells, further amplifying the tolerogenic effects of HLA‐G in tumor immune escape.

## Intercellular transfer of HLA‐G through extracellular vesicles

Extracellular vesicles (EVs) are lipid bilayer membrane vesicles with different sizes ranging from 30 to 10 000 nm in diameter and contain different panel of biomaterials such as proteins, lipids and nucleic acids. EVs can be secreted by almost all cell types including cancer cells.^47^ However, their different cell of origin makes EVs highly heterogeneous. Hitherto, four subtypes of EVs have been recognised based on their size: exosomes (70–150 nm), microvesicles (100–1000 nm), apoptotic bodies (> 500 nm) and oncosomes (> 1000–10 000 nm).^48^ Functionally, it is now well established that EVs can affect many aspects of physiological and pathological processes such as immune responses through intercellular transfer of their biomaterial content.^49^

In the context of cancers, the first evidence for an HLA‐G1 isoform transported through EVs *in vitro* was found in the supernatants of an HLA‐G1^+^ melanoma cell line (Fon) and an HLA‐G1‐transfected melanoma cell line (M8‐HLA‐G1).^50^ In clinical settings, a larger ubiquitinated HLA‐G complex with a higher molecular weight (50–150 kD) was found in exosomes from cancer patient ascites and pleural exudates *in vivo*.^51^ However, the types of HLA‐G isoforms remain unclear. The non‐typical molecular weight of HLA‐G might be caused by complex ubiquitination, such as different units of ubiquitin binding to proteins, and different protein structures, such as full‐length HLA‐G5 with α1, α2 and α3 extracellular domains and HLA‐G6, which lacks α2 extracellular domain.^52^ All of these factors could affect the different molecular weights of the ubiquitinated HLA‐G. In breast cancer patients treated with neoadjuvant chemotherapy, higher levels of sHLA‐G in peripheral circulating EVs were found to be positively related to disease progression of non‐metastatic but locally advanced breast cancer patients. Additionally, the higher levels of sHLA‐G in EVs are associated with the presence of stem‐cell‐like circulating tumor cells.^53^ In colorectal cancer (CRC) patients, we recently found that levels of sHLA‐G in exosomes derived from plasma were significantly higher than those in healthy controls, colorectal polyps and inflammatory bowel disease patients. Exosomal sHLA‐G was significantly associated with patient tumor status, lymph node metastasis and AJCC TNM stage. ROC analysis of the data also revealed that the diagnostic value of sHLA‐G in exosomes was superior to that of CEA and CA19‐9 for CRC patients.^54^ In an *in vitro* study, Grange *et al*.^55^ reported that higher levels of sHLA‐G‐bearing EVs were observed in CD105^+^ renal cancer stem cells than in CD105^−^ non‐tumor‐initiating renal cancer cells. Moreover, sHLA‐G‐bearing EVs derived from both CD105^−^ non‐tumor‐initiating renal cancer cells and CD105^+^ renal cancer stem cells can impair peripheral monocyte‐derived DC maturation and T‐cell activation through the immune inhibitory functions of HLA‐G. In addition, the immune‐suppressive power of sHLA‐G‐bearing EVs derived from CD105^+^ renal cancer stem cells is much higher than that from CD105^−^ renal cancer cells. These findings indicated that sHLA‐G in EVs might be derived from stem‐cell‐like cancer cells.

To date, only a few studies have been carried out on the clinical relevance of exosomal HLA‐G expression in tumors. However, much progress has been achieved in the study of tumor cell‐derived exosomes carrying PD‐L1.^56^ The data showed that exosomal PD‐L1 can inhibit the proliferation of CD8^+^ T cells, reduce the number of T cells in the spleen and lymph nodes and promote tumor growth.^19^ As mentioned above, exosomal HLA‐G levels have been observed to be related to the disease progression of non‐metastatic but locally advanced breast cancer patients and CRC patients.^53^, ^54^ It can be speculated that HLA‐G contained in exosomes functions similarly to both tumor cell surface expressed or secreted HLA‐G molecules, which interact with the receptors ILT2 and ILT4 expressed on various immune cells and transduce inhibitory signals. The immune suppression induced by HLA‐G favors tumor cell escape from host antitumor immune responses.^2^

## Intercellular transfer of HLA‐G through tunnelling nanotubes

Tunnelling nanotubes (TnTs) are non‐adherent, long, actin‐driven cytoplasmic protrusions that function as intercellular communication channels between adjacent and distant cells.^26^ The formation of TnTs can be generated by two ways: the ‘protrusion elongation’ manner driven by polymerisation of actin and the ‘cell dislodgement’ manner when cells initially in contact draw out nanotubes as they separate from each other.^57^ TnTs have distinctive characteristics that they connect at least two cells, contain F‐actin and do not touch substrate. Moreover, TnTs can transfer cellular cargo between neighbouring cells.^57^, ^58^ However, our understanding of TnTs is in its infancy, and tremendous aspects of TnTs remain to be explored, although TnTs have been investigated in a wide variety of non‐cancer cells and malignant cells.^59^ Among them, TnTs have been reported in immune cells such as B cells, T cells, NK cells, dendritic cells and macrophages and in cancer cells of different origin.^60^

In the context of cancer cells, particularly in malignant pleural mesothelioma, Lou and colleagues have performed comprehensive studies on TnTs in malignant cells both *in vitro* and *ex vivo* and, more recently, *in vivo* in murine models.^61^ In a series of studies, an *in vitro* modified wound‐healing assay revealed that TnT formation is along the invasive leading edge of a proliferating and migrating mesothelioma cell.^62^ Additionally, TnTs have been observed *in vivo* in resected mesothelioma and lung adenocarcinoma lesions, and in a murine model of osteosarcoma and human ovarian adenocarcinoma.^63^ In addition to these findings, a growing body of literature has indicated that TnTs play important roles in cancer pathogenesis, progression and resistance to therapy.^61^

Change in HLA antigen expression is very common in cancers, which is of critical significance in cancer initiation and progression and in the effects of therapy.^64^ However, evidence on the intercellular transportation of HLA antigens through TnTs in cancers is limited. In an earlier study, GFP‐tagged HLA‐Cw6 protein expressed on 721.21 cells could be transported to peripheral blood NK cells by the nanotubular highway.^65^ In another study, Schiller and colleagues^12^ reported that intercellular transfer of surface‐bound HLA‐A2‐EGFP fusion protein through TnTs has been observed in cells of myeloid origin and HeLa cells, indicating that HLA antigens can be exchanged between APCs through TnTs. Notably, no such phenomenon has been found for the soluble HLA‐G1 (HLA‐G1s)‐EGFP fusion molecule, which lacks the transmembrane region and a cytoplasmic tail. Based on this finding, we can speculate that the transmembrane region and cytoplasmic tail of HLA antigens are related to TnT transportation. In addition to the difference in the transmembrane region and the cytoplasmic tail between the surface‐bound HLA‐A2‐EGFP and HLA‐G1s‐EGFP fusion molecules, several factors may account for the inability of the HLA‐G1s‐EGFP fusion molecule to be intercellularly transferred through TnTs. First, the fused EGFP‐tag could interfere with the protein fold, which can lead to a misfolded molecular structure.^66^ Second, the EGFP‐tag may prevent the correct conformation of the molecule and the association of the proper chaperone required for HLA‐G expression.^67^ Third, the tagged or even the naturally expressed truncated HLA‐G isoforms could be retained in the endoplasmic reticulum.^68^ Therefore, given that multiple isoforms of HLA‐G can be generated, the different naturally expressed HLA‐G isoforms, including membrane‐bound and sHLA‐G isoforms, should be investigated in future studies.

## Translational implications of the intercellular transfer of HLA‐G

The immune‐suppressive functions of HLA‐G in tumor immunology have been well recognised since the past three decades. In addition to well‐defined immune checkpoints such as PD‐1/PD‐L1 and cytotoxic T lymphocyte‐associated protein 4 (CTLA‐4)/B7, the interaction between HLA‐G and its receptors ILT2 and ILT4 is emerging as a novel immune checkpoint.^11^

**Figure 2:**
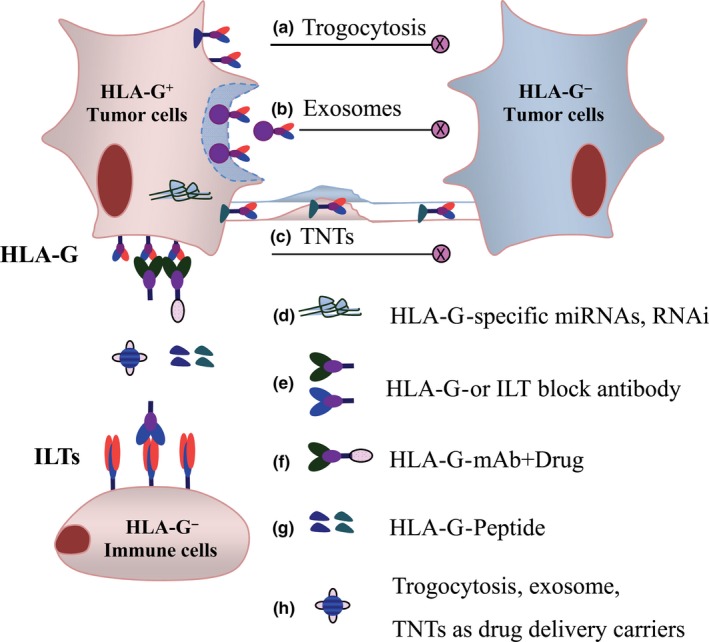
Multiple levels of HLA‐G‐targeted cancer immunotherapy strategies. Using (**a**) potential inhibitors to impair the formation and process of trogocytosis, (**b**) exosomes and (**c**) tunnelling nanotubes, which transport HLA‐G to other cells, (**d**) post‐transcriptional RNA interference to down‐regulate HLA‐G transcription, (**e**) specific blocking antibodies to block either HLA‐G or receptor ILTs, (**f**) HLA‐G antibodies conjugated with antitumor drugs to target HLA‐G‐positive cancer cells, (**g**) HLA‐G‐derived immunogenic peptides to activate the T‐cell immune response and (**h**) target HLA‐G^+^ tumor cells to develop drug delivery systems.

Given the various mechanisms of HLA‐G in tumor immune evasion, multiple levels of HLA‐G‐based therapeutic strategies should be considered, such as inhibition of the intercellular transfer of HLA‐G, down‐regulation of HLA‐G expression, blockade of the HLA‐G and receptor interaction and application of tumor cell‐expressed HLA‐G as a target to develop drug delivery systems (Figure 2).

First, inhibition of the intercellular transfer of HLA‐G could be obtained by various inhibitors involved in impairing the formation and production of trogocytosis, exosomes and TnTs. The process of trogocytosis was reported to be inhibited by inhibitors of mitogen‐activated protein kinase (PD98059) and protein kinase C (Rottlerin).^69^ The biogenesis and release of exosomes could be impaired by dimethyl amiloride and inactivation of the small GTP‐binding protein ARF6, respectively.^70^, ^71^ Additionally, the formation of TnTs could be inhibited by mTOR signalling inhibitors metformin and everolimus, and cytoskeletal inhibitors docetaxel and latrunculin A as previously reported.^72^, ^73^ Second, post‐transcriptional RNA interference by HLA‐G‐specific miRNAs (such as miR‐148, miR‐152, miR‐548q and miR‐628‐5p) has been demonstrated to reduce HLA‐G expression and recover NK cell lysis of tumor cells.^74^ Third, the masking of either HLA‐G or receptor ILTs with specific blocking antibodies could be a promising strategy for HLA‐G/ILTs‐based target therapy. In this context, a large body of research has demonstrated that blockade of HLA‐G and ILTs could restore the cytotoxicity of NK cells.^75^ For T cells, HLA‐G‐derived peptides HLA‐G^146–154^ and HLA‐G^26–40^ could effectively induce peptide‐specific CD8^+^ T cytotoxicity and tumor‐reactive CD4^+^ T‐cell responses, respectively, rendering the possibility of HLA‐G peptide‐based tumor immunotherapy.^76^, ^77^ Finally, targeting tumor‐specific HLA‐G to develop drug delivery systems has been carried out. An HLA‐G antibody conjugated to the surface of methotrexate (MTX)‐loaded nanobubbles (mAb_HLA‐G_/MTX/PLGA NBs) could be specifically transported to the HLA‐G‐positive choriocarcinoma cancer cells JEG‐3 *in vitro* and tumor tissues *in vivo* in a tumor‐bearing murine model of BALB/c (nu/nu) nude mice established with JEG‐3 cells. Data revealed that the HLA‐G‐targeted mAb released from the NBs could kill the residual JEG‐3 cells and inhibit the reoccurrence of tumors.^78^ Moreover, a recent study by Guo *et al*.^79^ reported that, using M1 macrophages as live‐cell carriers, doxorubicin‐loaded M1 macrophages (M1‐Dox) had a natural tropism to the ovarian cancer SKOV3 cells and delivered the drug doxorubicin very fast and accurately to the target cells through a tunnelling nanotube pathway. The authors also found that multiple organ metastases (liver, kidneys, spleen, gastrointestinal tract, diaphragm and uterine appendages) in the BALB/c (nu/nu) nude mice established with SKOV3 cells were reduced to nearly undetectable levels in the M1‐Dox‐treated group. To HLA‐G‐positive cancer cells, anticancer drugs in carriers conjugated an HLA‐G mAb can be guided and delivered precisely to kill HLA‐G‐positive tumor cells.

## Conclusion

Cancer cells have developed different effective and efficient strategies to escape immune surveillance and destruction by the host adaptive and innate immune systems. Altered expression and function of HLA antigens on tumor cells are of particular importance. Among them, HLA‐G is a molecule that is aberrantly induced and frequently detected in a wide variety of cancers, and its expression is correlated with unfavorable clinical outcome.^11^ Functionally, HLA‐G can bind its inhibitory receptors such as ILT2 and/or ILT4 expressed on various immune cells (NK cells, T cells, B cells, DCs, neutrophils) to inhibit the functions of these immune effectors, and bind the receptors expressed on Tregs and MDSCs to induce expansion of these regulatory cells.^2^ Consequently, HLA‐G‐mediated immune inhibition can collectively favor cancer cell immune escape and cancer development.

However, no tumor cell is an isolated island that acts autonomously, but complex networks of intercellular communication share transcriptomic and proteomic features continually among neighbour and distant cells.^13^ The basic and clinical significance of intercellular communication is increasingly acknowledged as being critical to tumoral morphologic heterogeneity, immune escape, tumoral metastasis, and recurrence and resistance to therapy.^33^ Intercellular transfer through trogocytosis, exosomes and even TnTs has been documented in a number of studies. Based on these reports, the unidirectional and bidirectional intercellular transfer of HLA‐G can occur from tumor cells to tumor cells, from tumor cells to immune effectors and from immune cells to immune cells, which can play critical roles in tumor advancement. Furthermore, previous findings showed a synergistic effect of exosomes on TnT transfer in cancer, indicating that these different intercellular transfer pathways are not mutually exclusive but rather very coordinated and complementary.^80^

Cancers are a very heterogeneous mixture of cells with distinct morphologies and phenotypes, such as intertumor and intratumor heterogeneity of HLA‐G expression, which has been addressed in a variety of cancers.^2^ Given that HLA‐G is a potent immune suppressor, in addition to directly inhibiting the function of immune effectors, the intercellular transfer of HLA‐G among tumor cells and immune cells extends its immune‐suppressive functions to a much broader spectrum.

First, through intercellular transfer, HLA‐G^−^ tumor cells can uptake HLA‐G molecules from adjacent HLA‐G^+^ tumor cells in a cell‐to‐cell contact‐dependent manner by trogocytosis, and TnTs transfer or can uptake the HLA‐G antigen derived from distant HLA‐G^+^ tumor cells carried by exosomes. As a consequence, the HLA‐G^−^ tumor cells turn into HLA‐G^acq+^ tumor cells. A few HLA‐G^+^ tumor cells can share and spread their HLA‐G to a larger population of HLA‐G^−^ tumor cells, thus, conferring extra protection to the cells that originally did not express HLA‐G. Second, different immune cells, such as NK cells, B and T lymphocytes and DCs, can acquire HLA‐G antigens from HLA‐G^+^ tumor cells, which can immediately convert the effector cells into temporary regulatory cells. Moreover, intercellular transfer of tumor cell‐derived HLA‐G among immune cells can also extensively amplify its immune suppression effects, leading to a broader tumor cell population resistance to antitumoral immunity.

In summary, increasing evidence has revealed that, in addition to HLA‐G expressed on tumor cells, intercellular transfer of HLA‐G among different types of tumor cells and immune cells plays important roles in the regulation of immune responses and in enriching cancer cell immune evasion strategies. However, a better understanding of the underlying molecular mechanisms and precise functions of these intercellular transfers will help us to translate this knowledge into therapeutic applications.

## Conflict of interest

The authors declare no conflict of interest.
